# Short-term factual recall, presence, and visio-vestibular discomfort in a 360° augmented-virtuality classroom environment: A feasibility study

**DOI:** 10.1371/journal.pone.0356395

**Published:** 2026-08-21

**Authors:** Kelly D. Carrasco, Andre M. Escarzaga, Melanie Gonzalez, Andrew Rosenfelder, Nicolas Beltran, Cory Ford, Kai Miguel, Leah C. A. Scott, Andres Carrasco

**Affiliations:** Department of Psychology, California State University, Fresno, California, United States of America; New York University Abu Dhabi, UNITED ARAB EMIRATES

## Abstract

As the field of virtual reality continues to advance, approaches that integrate naturalistic immersive content with artificial simulations have gained increasing scholarly attention in educational psychology, offering novel opportunities to assess learning within realistic yet experimentally controlled environments. The present pilot study evaluated the feasibility of a chroma-key augmented-virtuality (AV) system as a research and teaching tool, examining short-term factual recall, presence, and visio-vestibular discomfort among university students (n = 104) attending a prerecorded 360° AV lecture. In this system, participants were incorporated via chroma-key passthrough into a recorded classroom scene, thereby situating them physically within the virtual environment. Pre- and post-tests assessed immediate recall of lecture content alongside self-report measures of presence and comfort. Analyses revealed statistically significant improvements in immediate recall following the lecture (64.58% relative increase, *p* < 0.001, Cohen’s *d* = 0.88), although absolute post-test performance remained modest (M = 4.29 of 10 items). Participants reported moderate levels of presence (M = 3.50, SD = 0.72, on the 0–6 IPQ scoring) and minimal cybersickness symptoms (M = 2.12, SD = 0.92, on the 7-point CSQ-VR scale), and presence was not significantly associated with recall. Collectively, these findings indicate that short, passive 360° AV lecture formats constitute feasible tools for investigating short-term factual recall and user experience within controlled classroom contexts.

## Introduction

Over 30 years ago, Milgram and Kishino [1,2] conceptualized the *virtuality continuum* as a spectrum of multisensory experience. Within this framework, real-world and computer-generated elements are combined to give rise to distinct levels of experiential immersion and presence, from fully physical to entirely artificial. Virtual reality (VR) occupies the extreme end of the spectrum as a technology that immerses users in fully computer-generated three-dimensional environments. By simulating artificial spatial contexts, VR produces compelling sensations of presence and realism, enabling users to experience environments that are entirely detached from the physical world [1,3,4]. Although early attempts of immersive multimodal systems date back to the 1960s [5,6], recent technological developments have transformed these pioneering ideas into versatile applications across numerous domains. Specifically, in the last decade, greater accessibility of VR technology has facilitated its integration in mental health treatments [7–11], professional training [12–14], and the entertainment industry [15–17].

In educational research, VR has gained increasing recognition as a viable medium for instruction [18–20]. The immersive environments and spatial presence of VR offer new avenues for teaching and learning that extend beyond the confines of traditional classroom settings [13,21–23]. Examinations of VR as an academic tool have demonstrated its potential to enhance learning and content retention in science education [24–26], historical events [27,28], and mathematical concepts [29,30]. Despite these proven benefits, pupil interactions are frequently restricted to artificial computer simulations that lack the sensory detail and contextual authenticity of in-person educational environments [31,32]. These constraints have been shown to undermine the quality of the experience and discourage sustained engagement with VR systems [20,32,33].

Within the reality–virtuality continuum, mixed reality (MR) refers broadly to any environment in which real-world and virtual elements are presented together within a single display, that is, anywhere between the fully real and fully virtual extremes of the continuum [1,2]. MR encompasses a range of display types, including augmented reality (AR), in which virtual content is overlaid onto the user’s physical environment, and augmented virtuality (AV), in which real-world elements are embedded within a predominantly virtual scene. Unlike fully computer-generated VR, AV systems preserve real-world elements, such as the participant’s own body and immediate physical surroundings, within the virtual scene, maintaining physical grounding while delivering immersive virtual content [34–36]. This approach enables the development of immersive sceneries that are indistinguishable from the real world [4,36,37]. In addition to maintaining a strong sense of contextual realism, AV preserves the adaptability and control provided by digital media. In academic applications, this convergence offers an effective method of generating controllable and replicable environments that feel authentic and immersive to students [37–39].

Immersion and presence, although frequently used interchangeably, denote distinct constructs. Immersion refers to the objective technical characteristics of a system, such as display fidelity and field of view, that determine the sensorimotor contingencies the system is able to support. Presence refers to the subjective sense of being located within the mediated environment that such a system may evoke in the user [40]. A system of high technical immersion does not guarantee a correspondingly high sense of presence. In the sections that follow, immersion denotes the objective features of the technology, whereas presence denotes the experiential state it may produce. The evidence linking immersion and presence to learning, however, is mixed. Some studies report that more immersive systems and stronger presence are associated with better learning performance and spatial memory [22,41–44], whereas others find no relationship [45] or even reduced learning under high-presence conditions, potentially because rich immersive detail diverts attentional resources away from instructional content [26,46]. Most of this evidence, moreover, derives from interactive, computer-generated VR. It therefore remains unclear whether, in passive, low-interactivity AV classroom environments, presence contributes to factual learning or merely enriches the subjective experience without a corresponding cognitive benefit. The present study was designed in part to address this unresolved question.

Despite growing interest in AV environments as immersive educational tools, and notwithstanding the extensive literature on cybersickness in VR [47–49], relatively little empirical research has examined learning outcomes and visio-vestibular discomfort specifically in passive, low-motion, real-world classroom environments delivered through AV passthrough head-mounted displays [37,50]. Specifically, most studies have focused on low and high immersion computer-generated virtual environments, leaving questions about how real-world AV classroom environments impact students’ physical discomfort and learning outcomes largely unanswered. Furthermore, although fast-paced and user-controlled VR environments have been associated with symptoms of nausea, dizziness, and disorientation, collectively known as visual-vestibular conflict or cybersickness, the extent to which passive, low-motion simulations in educational contexts contribute to such responses remains unclear [48,51,52]. Therefore, by assessing pre- and post-lecture performance alongside self-reports of user experience, the present study aimed to evaluate the feasibility of AV for supporting short-term factual recall and its adequacy as a research and teaching tool. Two hypotheses guided this investigation. First, we predicted that students would exhibit significant improvement in immediate post-lecture recall relative to baseline. Second, we hypothesized that students would report elevated presence and low levels of discomfort, reflecting a positive and well-tolerated experience within the AV environment. Given the mixed prior evidence, the relationship between presence and recall was examined on an exploratory basis, without a directional prediction.

## Materials and methods

### Participants

Undergraduate students (n = 104; aged ≥18 years; normal or corrected-to-normal vision) were recruited via the university’s research participation platform to take part in a study of learning outcomes in an augmented-virtuality classroom. Data were collected between February 21, 2025 and April 30, 2025. All participants provided written informed consent and received course-related research credit. The study was approved by the California State University, Fresno Institutional Review Board (IRB protocol #2683) and was determined to involve minimal risk. All procedures were conducted in accordance with institutional policies and the Declaration of Helsinki. All personally identifiable information was removed prior to analysis.

### Measures

Presence and physical discomfort levels were assessed using the Igroup Presence Questionnaire (IPQ) and the Cybersickness Questionnaire (CSQ-VR), respectively. Immediate recall of lecture content was measured using multiple-choice tests. All questionnaires and assessments were conducted digitally using the Qualtrics software platform.

#### Presence.

The IPQ was used to assess the subjective sense of presence within the AV environment. The IPQ is composed of three subscales: Spatial Presence (five items assessing the perceived sense of physical location within the environment), Involvement (four items measuring attentional focus and engagement), and Experienced Realism (four items evaluating the subjective realism of the environment). Additionally, a single item is used to assess general presence. All items are rated on a 7-point Likert scale ranging from −3 (“fully disagree”) to +3 (“fully agree”) with higher scores indicating a stronger sense of presence. For analysis and figure presentation, responses were recoded to a 0–6 scale with a midpoint of 3, consistent with common IPQ scoring conventions. All IPQ descriptive statistics reported below use this coding. Negatively worded items were reverse-scored so that higher values on every item denote greater presence. The presence score comprises the five spatial presence items together with the general presence item, and the involvement and realism scores comprise their four items each. The IPQ total was computed as the mean of the three subscale scores. Internal consistency of the full scale was acceptable (Cronbach’s α = 0.74). Research has demonstrated the scale’s sensitivity to factors such as exploratory behavior, environmental predictability, and interactivity, offering strong support for its construct validity and utility in assessing presence in immersive virtual environments [53,54].

#### Cybersickness.

The CSQ-VR was used to evaluate physical discomfort resulting from exposure to the AV environment. This measure assesses nausea, disorientation, and oculomotor discomfort through six items rated on a 7-point Likert scale ranging from 1 (none) to 7 (severe). Participants rated the extent to which they experienced symptoms such as gastrointestinal discomfort, spatial disorientation, and visual strain. The CSQ-VR has demonstrated strong diagnostic validity and reliability in measuring cybersickness within immersive virtual reality settings, supporting its use as an effective tool for assessing adverse physical responses in immersive instructional research contexts [55,56].

#### Short-term factual recall.

Content knowledge was assessed using 10-item multiple-choice tests, each with four response options, administered before and after the AV lecture. To control the potential impact of the tests order effects and minimize test-retest bias, participants were randomly assigned to one of two counterbalanced test versions. Questions were derived from and limited to the lecture content. The lecture was designed to introduce core psychological concepts related to grief, including theoretical models, emotional processes, and applied support strategies. Test items were developed directly from verbatim lecture transcripts to ensure close correspondence with instructional content. The assessment was primarily designed to measure short-term factual recall and was not intended to evaluate long-term retention or transfer of conceptual knowledge. Both test versions were reviewed by faculty with expertise in school psychology and neuroscience for content accuracy and clarity.

### Learning design

The AV lecture was designed as a curriculum-integrated instructional session on the psychological stages of grief, a topic selected for its defined factual content and suitability for a single-session exposure. The lecture followed a traditional didactic format: an experienced professor delivered the content from behind a podium without visual aids or graphical projections, replicating the structure of a conventional university lecture. This design choice was deliberate, as the study aimed to evaluate AV as a delivery medium for standard instructional content rather than as a platform for interactive or simulation-based pedagogy. To enhance ecological validity, naturalistic classroom distractors (e.g., late-arriving students, side conversations, mobile device use) were scripted and introduced at approximately 30-second intervals, simulating realistic attentional demands. The 10-item multiple choice assessment was developed in alignment with the specific factual content of the lecture, with all questions derived directly from the material presented. Two counterbalanced versions were created to control for order effects. This design situates the study as a feasibility investigation of AV-delivered instruction under conditions approximating a real-world classroom, rather than a test of an optimized or interactive AV learning intervention.

### Procedures

After arrival at the experimental room, participants were seated at a computer workstation and presented with an on-screen information form describing the study procedures and stating their right to withdraw from the experiment at any time without penalty. After reviewing the document, participants provided digital written informed consent. A pre-lecture multiple choice test was administered to establish a baseline measure of familiarity with the lecture content. Participants were then fitted with a headset and immersed in the pre-recorded AV lecture. After the session ended, participants completed a post-lecture-test to assess content retention and subjective experiences using the IPQ and CSQ-VR. The procedure concluded with a brief demographic questionnaire that collected information on age, gender, and prior experience with virtual reality technology. The entire process took approximately 40–50 minutes per participant.

### Apparatus and stimuli

#### Lecture production.

A 15-minute-long lecture about the psychological stages of grief was delivered by an experienced professor and recorded with a 360° video camera (Insta360 X4, 5.7K HDR, 60 fps) and a NT-SF1 3D ambisonic condenser microphone (RØDE) attached to a F6 multitrack field recorder (Zoom). The recording devices were placed on the third row of a lecture hall, approximately 3 meters away from the professor and angled at 15° from the line of sight at the podium. The resulting 360° footage was monoscopic (non-stereoscopic) and was therefore presented in the headset without binocular depth cues. The professor did not use visual aids or graphical projections and remained stationary behind the podium for the entirety of the lecture, limiting visual motion within the scene to reduce potential contributions to cybersickness. To maintain the realism of the AV classroom environment, typical classroom distractors were incorporated, including students arriving late, engaging in conversation during the lecture, and using mobile devices. These events were embedded within the ongoing classroom scene, with the instructor continuing to lecture from the podium, and they differed in duration and in their location within the lecture hall. No auditory or visual cue signaled an impending event. Although the average inter-event interval was approximately 30 s, the exact onset of each event varied within a time window rather than occurring at fixed intervals, reducing the likelihood that participants could anticipate the timing of upcoming events. Student-actors were current university undergraduates and were directed to act naturally and maintain a realistic classroom experience.

#### Lecture delivery.

**Fig 1:**
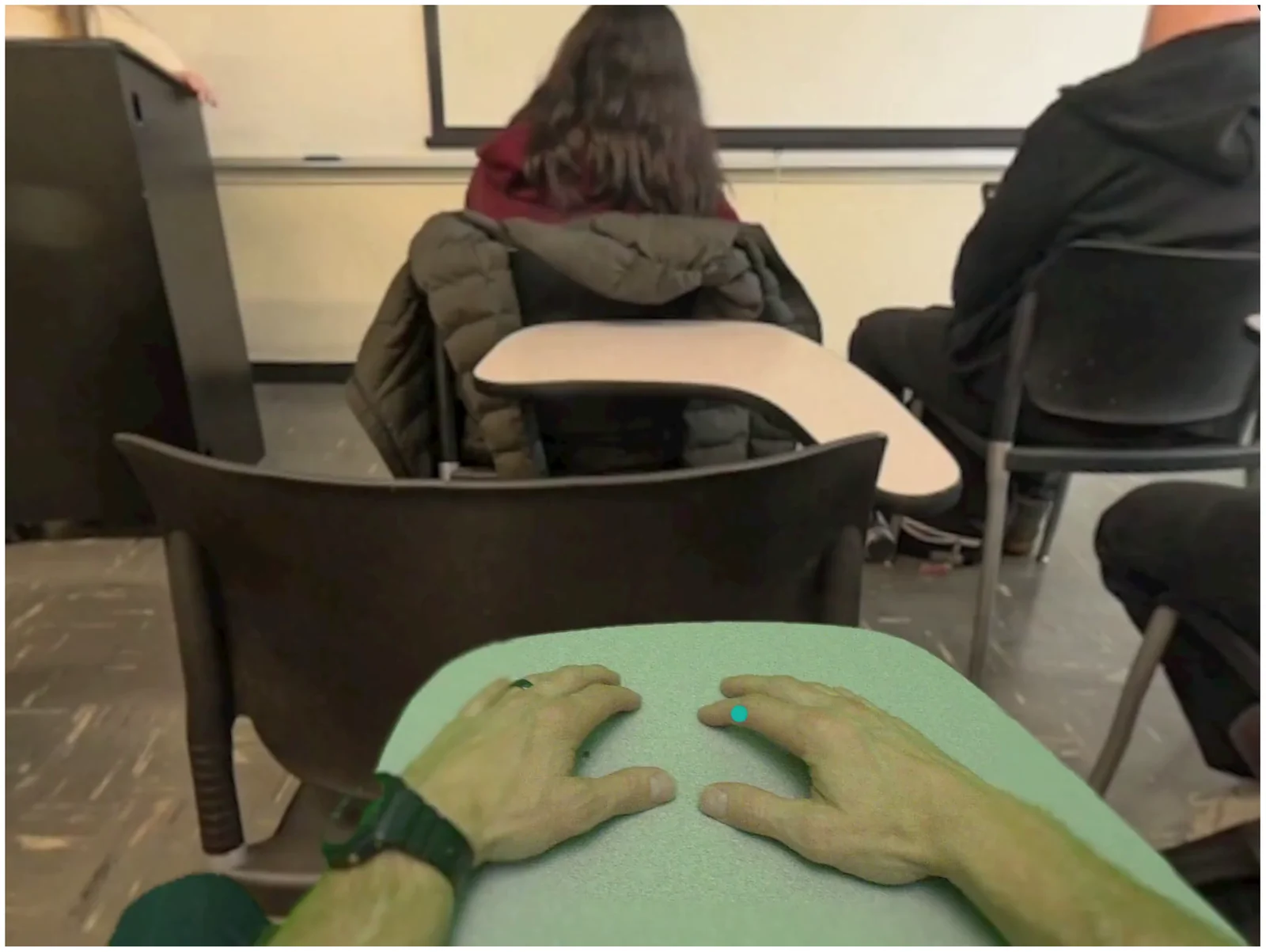
In-headset view of the composited augmented-virtuality classroom. A representative frame of the in-headset view, showing real physical elements (the wearer’s hands, arms, and the desk surface) embedded within the prerecorded 360° virtual lecture hall. The virtual scene supplies the seated students, the desks, the podium, and the room architecture, while the wearer’s hands, forearms, and desk are passed through from the physical laboratory and composited into the scene in real time. The frame was recorded during a demonstration session with a member of the research team and reproduces the view presented to participants.

The study was conducted in a purpose-built chroma-key (CK) augmented-virtuality laboratory. The experimental room (3.23 m × 2.77 m × 3.00 m) was coated with uniform green paint on all visible surfaces, including walls, floor platform, door fixtures, and base moldings, to enable reliable color-keying across the headset’s full field of view. Two LED flood lights were mounted behind the participant position to maintain consistent wall illumination and minimize keying artifacts. Participants were seated at a standard classroom desk positioned at the center of the CK room. They were fitted with a Varjo XR-4 mixed-reality headset (Varjo Technologies, Helsinki, Finland) and a set of headphones (Sennheiser HD 300). The Varjo XR-4 features dual mini-LED displays (3,840 × 3,744 pixels per eye, 90 Hz) and stereo passthrough cameras that enable real-time compositing of the physical environment with virtual content. A calibration procedure (~5 minutes) was conducted to optimize system alignment. Following calibration, the 360° 15-minute lecture was presented within the AV environment. In the present study, the green-painted surfaces were digitally replaced in real time by the prerecorded 360° classroom video, while participants’ physical bodies and desk, which were not green, remained visible within the composited scene. This arrangement situated each participant physically within the recorded classroom environment. Their own body appeared within the virtual lecture hall, constituting an augmented-virtuality experience in which real-world elements were embedded within a predominantly virtual scene: the headset’s high-resolution cameras captured and rendered the participant’s physical surroundings in real time, while the pre-recorded 360° lecture content was composited into the chroma-keyed regions of the visual field. This configuration preserved perceptual access to real-world visual anchors (e.g., the participant’s own hands, the desk surface) while simultaneously immersing participants in the recorded lecture space, thereby situating the experience within the AV portion of the reality-virtuality continuum rather than in fully occluded VR. Thus, at all times, participants could simultaneously see (a) the prerecorded virtual lecture hall, including the professor, other students, and the room architecture, and (b) the real physical elements that were not chroma-keyed, namely their own body (hands, arms, torso, and legs) and the desk surface in front of them. The green-painted walls, floor, and door of the physical room were fully replaced by the virtual scene and were not visible during the lecture. A representative participant view of the composited AV scene is shown in Fig 1, and a video recording of the experimental setup, showing a member of the research team seated within the chroma-key arena with the composited in-headset view displayed on the operator monitor, is available in the project’s OSF repository (https://osf.io/gby58).

### Data analysis

Data analyses were conducted using R-Studio (R Foundation for Statistical Computing). Descriptive statistics, including means and standard deviations, were calculated for self-report measures of presence (IPQ) and cybersickness (CSQ-VR) to characterize participants’ subjective experiences of engagement and physical comfort during the AV session. To evaluate changes in short-term factual recall, a paired-sample t-test was performed to compare pre- and post-lecture test scores. Cohen’s d and its 95% confidence interval were computed directly from the raw paired data as the effect size. In addition, descriptive analyses were conducted to examine measures of presence (IPQ), physical discomfort (CSQ-VR), and immediate recall. For all analyses, correlation coefficients (*r*), *p*-values, and 95% confidence intervals are reported. For non-significant effects, Bayes factors (BF01) were additionally computed using the BayesFactor package (version 0.9.12–4.7) in R with the package’s default prior to quantifying the relative evidence for the null hypothesis. Values greater than 3 were interpreted as at least moderate evidence for the null, and values between 1 and 3 as inconclusive. Correlational analyses were conducted for exploratory purposes and were not powered to detect small effect sizes. Findings should be interpreted with appropriate caution.

## Results

### Demographics

A total of 74 females (71.2%), 28 males (26.9%) and 2 participants (1.9%) that did not disclose their gender participated in the study. Ages ranged from 18 to 28 years (M = 19.70, SD = 1.93). Most participants (68.3%) reported prior experience with virtual reality, through gaming (41.8%), simulations (19.6%), educational applications (17.7%), social interaction (7.0%), and other uses (5.1%). A total of 8.9% did not provide information about former experience with VR. Normal vision was reported by 53.8% of participants, and 46.2% used glasses or contact lenses to achieve corrected-to-normal vision. A total of 43.3% of participants indicated never experiencing susceptibility to motion sickness, 30.8% experienced them rarely, 18.3% sometimes, and 7.7% often. Comfort with educational technology varied, 40.4% of participants reported feeling somewhat comfortable, 25.0% were neutral, 22.1% felt somewhat uncomfortable, 11.5% felt extremely comfortable, and 1.0% reported feeling extremely uncomfortable.

### Effect of augmented-virtuality instruction on short-term factual recall

**Fig 2:**
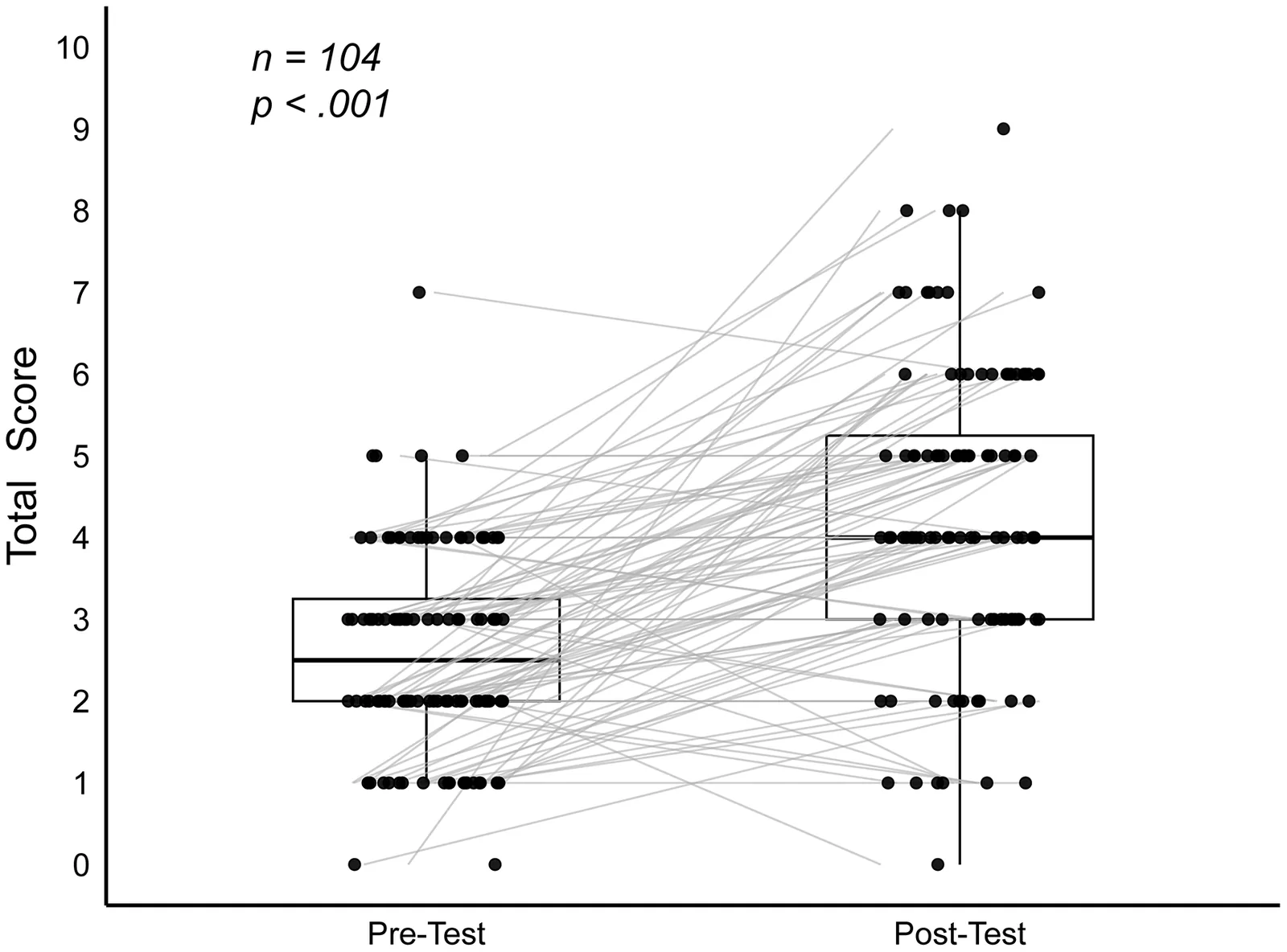
Test scores before and after an AV instructional session. Individual and summary test scores on a 10-item multiple-choice test (four response options per item) administered prior to (Pre-Test) and following (Post-Test) an AV instructional session. Boxes indicate the median and interquartile range, and whiskers indicate the range. Dots represent individual participant scores, and gray lines connect each participant’s paired pre- and post-test scores. A statistically significant elevation in post-test scores relative to pre-test baseline, t(103) = 9.0, *p* < .001, demonstrated a 64.58% relative improvement in mean performance, indicating that exposure to the AV instructional session was associated with substantial gains in immediate recall relative to baseline, although absolute post-test performance remained modest (M = 4.29 of 10). Notably, near-chance pre-test performance (M = 2.61 correct responses out of 10, chance = 2.5) confirms the absence of prior familiarity with the instructional content, thereby strengthening the interpretability of observed gains as a function of AV exposure rather than pre-existing domain knowledge. These findings support the utility of AV platforms for assessing immediate recall in short-form educational interventions.

Pre- and post-lecture assessments were administered to evaluate content knowledge. Pre-test scores approached chance level (M = 2.61, SD = 1.24), indicating that, on average, participants had no meaningful prior knowledge of the lecture content. In contrast, post-test scores increased to M = 4.29 (SD = 1.80), t(103) = 9.0, *p* < .001, reflecting a 64.58% relative improvement (Fig 2). In absolute terms, however, mean post-test performance corresponded to 42.9% correct, indicating that participants recalled fewer than half of the tested content immediately after the lecture. This statistically significant gain demonstrates that exposure to the AV lecture was associated with enhanced short-term factual recall relative to baseline performance. The effect size for this improvement was large (Cohen’s *d* = 0.88, 95% CI [0.65, 1.11]). The mean pre–post improvement was 1.68 points, 95% CI [1.31, 2.05]. Given the single-group pilot design, these results should be interpreted as preliminary, as no comparison condition was included to isolate the contribution of the AV format.

### Presence

**Fig 3:**
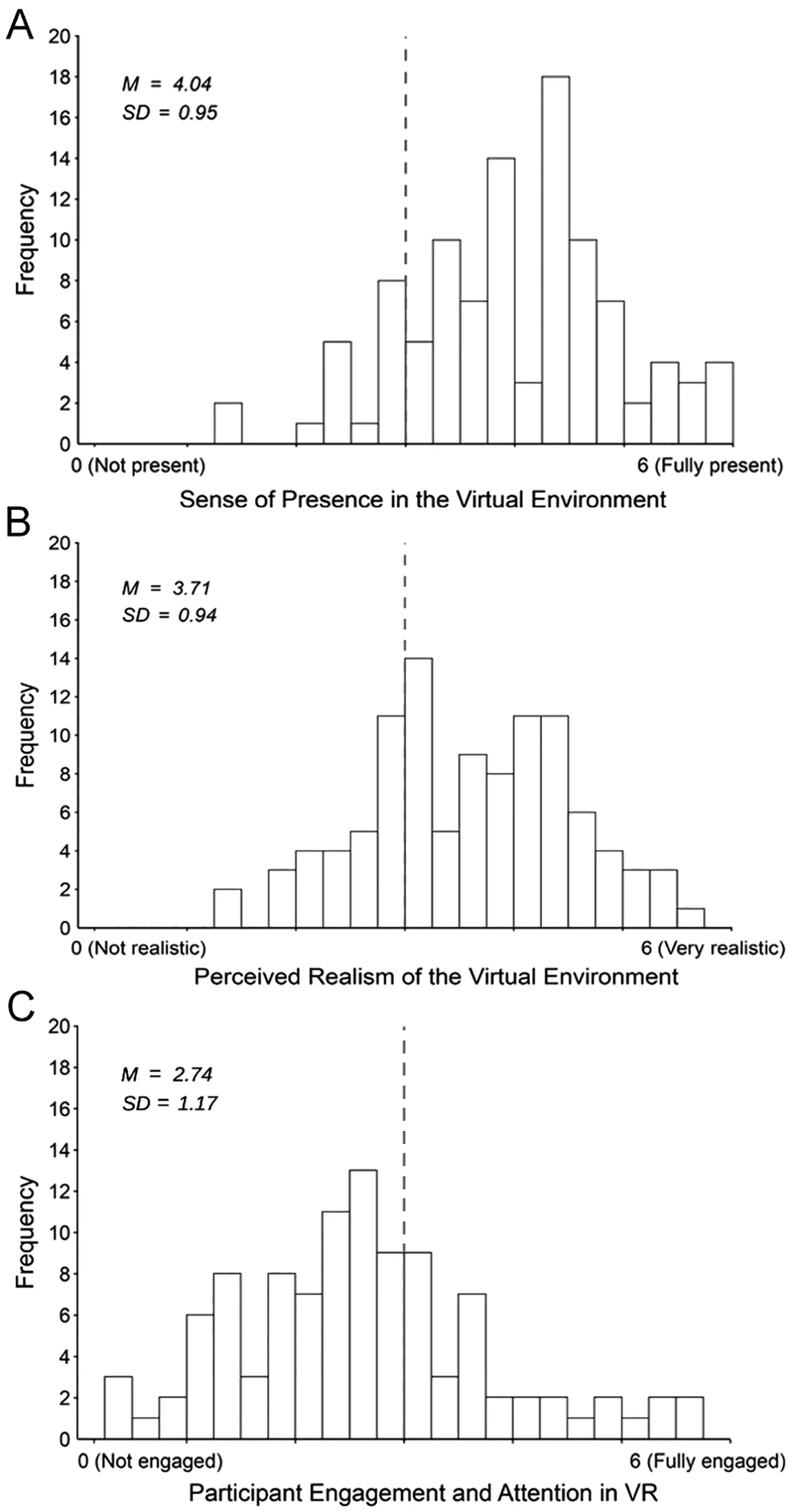
Subjective presence reported following the AV lecture. Scores are presented for the three subscales of the IPQ: (A) Presence, (B) Experienced Realism, and (C) Involvement. Participants reported moderate levels of presence (0–6 scoring; scale midpoint = 3), with the highest ratings observed for Presence (M = 4.04, SD = 0.95), followed by Experienced Realism (M = 3.71, SD = 0.94) and Involvement (M = 2.74, SD = 1.17). These ratings suggest that participants experienced the AV environment as perceptually convincing and engaging. Correlational analyses indicated no significant relationship between presence scores and post-test performance, suggesting that subjective presence did not directly influence immediate recall.

Participants’ subjective experiences within the AV environment were assessed using the IPQ. The overall mean score (M = 3.50, SD = 0.72, on the 0–6 scoring; scale midpoint = 3) indicated a moderate level of presence, falling somewhat above the scale midpoint. Subscale means diverged. Presence (M = 4.04, SD = 0.95; Fig 3A) and experienced realism (M = 3.71, SD = 0.94; Fig 3B) fell above the scale midpoint, whereas involvement (M = 2.74, SD = 1.17; Fig 3C) fell below it. This pattern indicates that participants located themselves within the virtual classroom and found it perceptually convincing, while remaining comparatively aware of their real surroundings.

A Pearson correlation analysis indicated no significant relationship between total IPQ scores and post-test performance, *r* = .04, *p* = .71, 95% CI [–.16,.23], BF01 = 7.61, indicating substantial evidence for the null and suggesting that participants’ sense of presence was not directly linked to immediate recall. These correlational findings should be interpreted with caution, as the study was not powered to detect small effect sizes. These findings highlight that while participants reported a moderate sense of presence, this subjective experience was unrelated to measurable differences in immediate recall.

### Cybersickness

**Fig 4:**
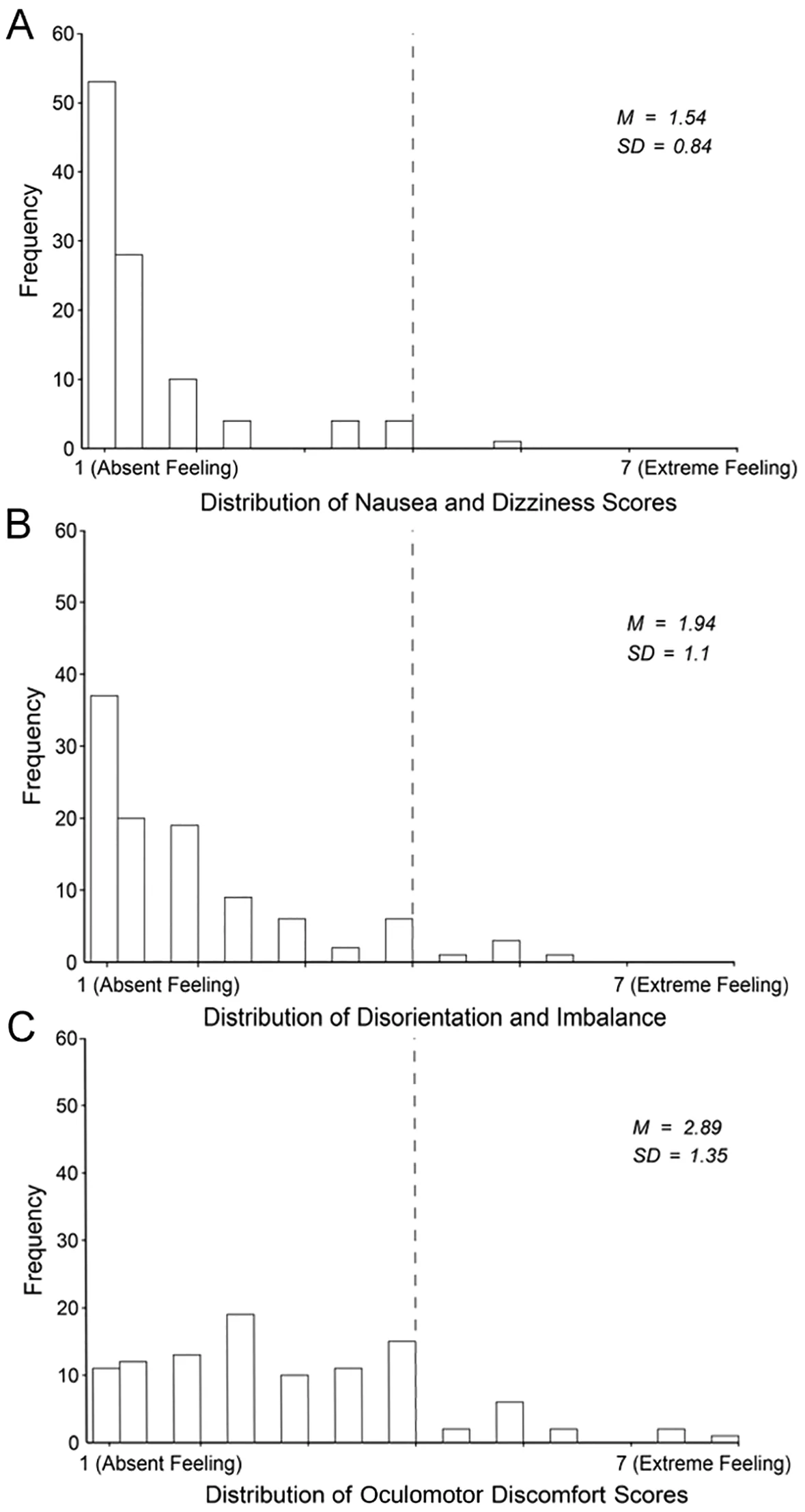
Cybersickness symptoms reported after the AV lecture. Scores are presented for the three dimensions of the CSQ-VR: (A) Nausea, (B) Disorientation, and (C) Oculomotor. Participants reported mild levels of physical discomfort, with the most reported symptoms related to the oculomotor subscale (M = 2.89, SD = 1.35), followed by Disorientation (M = 1.94, SD = 1.10), and Nausea (M = 1.54, SD = 0.84). The overall CSQ-VR score was M = 2.12 (SD = 0.92), reflecting tolerable symptom levels. A non-significant negative correlation between cybersickness severity and post-test performance (*r* = –.18, *p* = .07) suggests that physical discomfort did not meaningfully impair immediate recall.

Symptoms of nausea, disorientation, and oculomotor discomfort related to VR use were measured with the CSQ-VR scale. The overall CSQ-VR score was M = 2.12 (SD = 0.92), with mean scores of M = 1.54 (SD = 0.84, Fig 4A) for nausea, M = 1.94 (SD = 1.10, Fig 4B) for disorientation, and M = 2.89 (SD = 1.35, Fig 4C) for oculomotor symptoms. Oculomotor discomfort, including visual strain and fatigue, was reported most frequently, followed by disorientation symptoms such as dizziness and instability, and lastly nausea (Fig 4). A Pearson correlation analysis examining the relationship between total CSQ-VR scores and post-test performance revealed a weak, non-significant negative correlation, *r* = –.18, *p* = .07, 95% CI [–.36,.02], BF01 = 1.63. These findings demonstrate that, on average, participants experienced mild symptoms of cybersickness and that these symptoms were not significantly associated with immediate recall, although the Bayesian result was inconclusive (1 < BF01 < 3) and therefore does not affirmatively establish the absence of an association.

Together, these results demonstrate that participants experienced moderate levels of presence within the AV learning environment and achieved significant gains in immediate recall. While participants reported moderate presence and only mild oculo-vestibular discomfort, neither measure showed a significant correlation with recall performance.

## Discussion

The present study examined the feasibility of a 15-minute prerecorded 360° AV lecture for short-term factual recall, presence, and oculo-vestibular discomfort among university students. Data analyses detected a significant improvement in post-lecture scores, with moderate levels of presence and minor symptoms of cybersickness. However, given the single-group design and the absence of a comparison condition, these results should be interpreted as preliminary evidence of feasibility rather than as a demonstration of the unique efficacy of the AV format.

The magnitude of the observed gains should also be interpreted with caution. Although post-test scores improved by 64.58% relative to baseline, absolute post-test performance averaged only 4.29 of 10 items (42.9% correct), indicating that participants failed to recall the majority of the tested material immediately after the lecture. Several features of the design likely contributed to this modest absolute performance. The content was delivered in a single passive exposure at a fixed pace, and participants were unable to pause, review, or take notes. Scripted classroom distractors were deliberately embedded in the scene, and the assessment followed the lecture immediately, without opportunity for consolidation or restudy. Moreover, considering no comparison group received the same content through a traditional live lecture or a standard 2D video, the observed gains cannot be attributed specifically to the AV medium. They may reflect, in whole or in part, simple exposure to the material or practice effects associated with repeated testing. Accordingly, the pre–post improvement is best understood as evidence that the AV paradigm is feasible and sensitive to instructional exposure.

### Augmented virtuality in educational practice

The gains in immediate recall observed in the present study are consistent with prior research demonstrating that immersive technologies can enhance experiential learning when embedded within well-structured educational frameworks [13,25,57]. These findings highlight the potential of AV as an instructional tool for both traditional learners and those constrained by logistical, geographical, or financial barriers that require off-line educational content delivery. However, successful implementations of this educational approach will depend on how the physiological effects induced by immersive technologies are mitigated by educators. In particular, symptoms of cybersickness, such as eyestrain, dizziness, and fatigue, though mild in this study, have been shown in other contexts to negatively affect engagement and limit session duration [48,58]. Therefore, educators should design strategies that minimize abrupt motion and offer acclimatization protocols prior to exposure [59–61]. Furthermore, educational plans should aim to minimize distractions within AV environments to prevent the diversion of cognitive resources away from instructional objectives [13,62,63]. Specifically, instructional designers should consider balancing realism and interactivity with clarity and simplicity, ensuring that environmental features support, rather than distract from, educational content.

As AV technologies continue to advance, their integration into classroom and distance learning contexts may provide unique opportunities for equitable access to high-quality instruction. For example, while currently, AV-based instruction often relies on pre-recorded lectures or demonstrations, future integrations of this technology are likely to implement live-streamed AV environments, enabling students to interact in real time with instructors [64]. Such developments may closely imitate the pedagogical benefits of direct teacher-student interactions. The role of environmental design features in shaping learner satisfaction within VR contexts, further suggests that deliberate attention to persuasive design elements may amplify the engagement and comfort benefits observed in the present study [65]. More broadly, recent explorations of university students’ perceptions of metaverse-based virtual learning environments [66] indicate growing openness to immersive instructional modalities, positioning AV classroom environments within a wider landscape of next-generation educational technologies.

### Augmented virtuality in educational research

The present results support the potential use of AV-based instructional environments in educational research. In particular, the high degree of experimental control and perceptual immersion provided by AV minimizes environmental variability. Therefore, the use of pre-recorded lectures within AV paradigms can ensure that all participants experience an identical instructional environment, thereby eliminating confounding factors in educational studies such as differences in instructor delivery and classroom atmosphere [67]. Thus, by producing experimental replicability while preserving a sense of realism, AV holds the potential to overcome well-acknowledged methodological limitations of educational research [68,69].

### Presence and short-term factual recall in augmented-virtuality environments

Previous research has demonstrated that high levels of immersion are positively correlated with learning performance [22,41,42] and spatial memory [43,44]. However, the present study found no significant correlation between self-reported presence and post-test performance. This result suggests that greater presence does not inherently lead to increased immediate recall. While immersion can enrich subjective learning experiences by increasing engagement and presence, it may be insufficient to produce measurable gains in factual recall or conceptual understanding. This finding is consistent with the work of Kubr et al. [45], who reported that immersion did not always correlate with learning outcomes in VR environments. Moreover, other studies have shown that excessive immersion can hinder learning. For instance, Makransky et al. [46] observed that high-presence immersive VR simulations impaired learning outcomes.

Collectively, these findings indicate that the relationship between immersion and learning is not linear [45,46]. Immersion can enhance, have no impact on, or even hinder learning, depending on contextual factors such as task complexity, cognitive load, and user attention [20,26,45,61,62]. Research examining the role of persuasive design features in VR learning environments further suggests that design choices intended to heighten engagement may differentially affect learning satisfaction and knowledge acquisition, supporting the view that immersion-related design choices do not uniformly translate into learning gains [65]. Several theoretical frameworks help explain why presence and factual recall were unrelated in the present context. From a cognitive load perspective, the passive, fixed-pace format of the AV lecture ensured that instructional content was delivered to all participants equally, regardless of their level of felt presence [61,62]. Considering that participants were not required to navigate, make decisions, or interact with the virtual environment, the encoding of lecture content depended primarily on sustained attention to the verbally delivered material rather than on the depth of environmental immersion [26,45]. Higher levels of immersion may have intensified participants’ processing of peripheral features of the virtual classroom, including the scripted classroom distractors and the visual details of the recorded lecture hall, without channeling additional cognitive resources toward the instructional content itself [26,45]. Prior work has demonstrated that environmental complexity in immersive settings can divert attentional resources away from learning objectives, suggesting that the perceptual richness of an immersive environment may be dissociable from its instructional effectiveness [26,45,61].

A complementary account draws on the distinction between presence and learning as separable psychological outcomes [45,70]. While immersion may modulate the emotional quality and subjective authenticity of the experience, the encoding of declarative content may depend more on attentional focus and working memory engagement than on the sense of physical situatedness within the environment [26,61,62]. Together, these accounts suggest that in passive instructional formats, presence and factual learning may operate through partially independent pathways, and that the benefits of immersion for learning may be most pronounced in contexts requiring active exploration, problem-solving, or decision-making rather than reception of verbally delivered content [20,33,45,46].

The absence of interactivity in the present paradigm may be particularly important for interpreting the null presence–recall association. The AV lecture was entirely passive and fixed-paced, and participants could not pause, navigate, manipulate objects, or make decisions within the environment. Emerging work on immersive learning environments suggests that the benefit for learning depends less on immersion or presence per se than on whether learners engage in generative activity. Increases in immersion can raise presence and embodiment without improving transfer, and higher immersion has not consistently produced better memory retention [33,45], whereas the addition of a generative learning strategy to an immersive lesson improves learning outcomes [26]. Studies of interactive VR have also reported that visual discomfort can curtail engagement, particularly when users must actively move through the environment. In one comparison across levels of immersion, more than half of the participants navigating a virtual maze in a fully immersive head-mounted display discontinued the task before the allotted time, and nausea increased significantly relative to less immersive presentations [71]. Viewed against this literature, the present pattern of moderate presence, minimal visio-vestibular discomfort, and no presence–recall association is consistent with a passive, low-motion context in which presence had little opportunity to translate into learning-relevant cognitive engagement, while the same passivity kept discomfort low. Direct comparisons of passive and interactive AV formats will be needed to test this account.

Two features of the system further shape how these conclusions should be interpreted. First, although overall presence was moderate, the subscale means diverged (2.74 to 4.04 on the 0–6 scoring). Involvement, the dimension most closely tied to the attentional engagement that learning requires, fell below the scale midpoint. This raises the possibility that the passive format sustained a sense of being located within the classroom without sustaining attention to its content and that presence in this restricted sense was insufficient to influence recall. Second, the 360° lecture footage was monoscopic rather than stereoscopic, so the virtual scene was presented without binocular depth cues. Non-stereoscopic capture likely reduced the immersive fidelity of the system relative to stereoscopic 360° video and may thereby have limited the levels of presence that participants could attain. Future implementations using stereoscopic capture could test whether higher-fidelity depth information increases presence, and whether such increases have consequences for recall.

### Augmented-virtuality environments and physical comfort

Differences in immersive instructional design, in particular user movement and environmental motion, can impact user experience and learning outcomes. Immersive experiences that limit user locomotion and rely on visual scene changes (e.g., seated or passive viewing conditions) tend to reduce visual–vestibular mismatch, a principal contributor to cybersickness [48,49,72]. These low-motion environments promote a stable sense of presence while maintaining physical comfort, sustained attention, and cognitive engagement [49,57,61,62]. In contrast, immersive systems that involve active navigation or rapid shifts in virtual content enhance spatial presence but intensify cybersickness levels [47,51,73].

In the present AV system, participants’ physical bodies remained visible within the composited scene throughout the session. These real-world visual anchors, particularly the visibility of one’s own body and desk, may have contributed to perceptual stability by providing consistent spatial reference points that reduce the sensory conflicts associated with cybersickness [2,35]. This stands in contrast to fully occluded VR head-mounted displays, in which the external environment is entirely replaced, and no real-world anchors are available to stabilize the user’s perceptual experience [49]. Comparisons across immersion levels have demonstrated that static or semi-immersive systems yield higher levels of user tolerance [47,70,71].

In the present study, the passive, low-motion nature of the AV environment likely contributed to the mild symptoms of visual strain and fatigue. These results support previous investigations that did not detect a correlation between symptoms of cybersickness and learning outcomes [61,73], and highlight the importance of balancing perceptual immersion with physical comfort in AV instructional design. The integration of real-world visual cues within a stable AV framework, as achieved here through chroma-key passthrough compositing, may represent one approach to optimizing this trade-off, enabling immersive educational experiences without compromising user well-being. While the present study employed an AV passthrough configuration that preserved perceptual integration of physical and virtual elements, the absence of active user interaction with the virtual content places the experience toward the lower end of the AV spectrum. The classification of passive, low-interactivity experiences within the reality-virtuality continuum remains an open question in the field [1,2,4]. Future work should systematically examine how varying degrees of interactivity and real-world integration affect cybersickness, immersion, and learning outcomes across the continuum.

### Limitations and future research

The present study should be interpreted in light of several important limitations. First, the study employed a single-group design without a non-immersive comparison condition, which precludes causal inferences about the unique contribution of the AV format to the observed learning gains. Future work should incorporate active control conditions, such as traditional classroom instruction or standard video lecture formats, to isolate the specific instructional value of the AV approach. Second, the sample was composed entirely of undergraduate students recruited from a single university and was predominantly female (71.2%), limiting generalizability to other populations, age groups, and educational contexts. Third, the study was limited to a single subject matter domain (grief and bereavement) and a single passive instructional format. The 10-item multiple-choice test further captures primarily immediate factual recall and does not assess deeper conceptual transfer or application-based competencies. Further studies are needed to examine how different types of AV content, including interactive versus passive presentations, and different subject domains influence learning outcomes across academic disciplines. Emerging research on metaverse-based learning environments provides a useful comparative context for situating the present passive AV format and identifying directions for future development [66]. Fourth, the study involved a 15-minute lecture, which is notably shorter than standard class sessions. While this duration is consistent with evidence that cybersickness symptoms tend to increase with longer exposure durations, such that shorter immersive sessions are generally better tolerated [61], future research should examine whether longer exposure durations yield proportional gains or lead to diminishing returns due to fatigue, cybersickness, or attentional decline. Future research might also consider how variations in teaching style, including instructor enthusiasm, pacing, and communicative gesture, could differentially affect learner engagement and retention within immersive environments. In addition, the 360° footage was monoscopic, limiting the depth fidelity of the virtual scene. Fifth, cybersickness and discomfort were assessed through self-report measures alone, which capture subjective experience but cannot provide a full physiological profile of visio-vestibular conflict. Future investigations should supplement validated self-report instruments such as the CSQ-VR with objective indicators including electrooculography, postural stability assessments, or galvanic skin response. Finally, the correlational analyses examining relationships between presence, discomfort, and recall performance were exploratory and limited by sample size, reducing statistical power to detect small effects. Future studies should employ adequately powered designs with active control conditions and assessments capable of measuring higher-order learning outcomes, providing a more rigorous basis for evidence-based guidelines on the integration of AV into diverse educational settings.

## Conclusion

The results of this pilot study support the feasibility of chroma-key AV environments for examining short-term factual recall and user experience in controlled classroom contexts. While mild cybersickness symptoms were reported, they did not significantly affect recall performance. These preliminary findings suggest that with careful design and consideration of user comfort, AV can serve as both a practical instructional tool and a reproducible experimental framework in educational psychology research. Future work incorporating control conditions, longer exposure durations, and objective physiological measures will be needed to more fully evaluate the educational and methodological potential of this approach.
